# Electronic health record alerts enhance mass screening for chronic hepatitis B

**DOI:** 10.1038/s41598-020-75842-8

**Published:** 2020-11-05

**Authors:** Eric Chak, Chin-Shang Li, Moon S. Chen, Scott MacDonald, Christopher Bowlus

**Affiliations:** 1 Division of Gastroenterology and Hepatology, UC Davis School of Medicine, 4150 V Street, PSSB 3500, Sacramento, CA USA; 2 School of Nursing, The State University of New York, University at Buffalo, Buffalo, NY USA; 3 Division of Hematology and Oncology, UC Davis School of Medicine, Sacramento, CA USA; 4 Division of Clinical Informatics, UC Davis Medical Center, Sacramento, CA USA

**Keywords:** Disease prevention, Hepatitis B

## Abstract

To measure the effect of an electronic health record (EHR) alert on chronic hepatitis B (CHB) screening among at-risk Asian and Pacific Islanders (API). API patients who had not yet completed hepatitis B surface antigen (HBsAg) testing were identified by a novel EHR-based population health tool. At-risk API patients in Cohort 1 (primarily privately insured) and Cohort 2 (includes Medicare and/or Medicaid) were randomized to alert activation in their electronic medical charts or not. In total, 8299 API were found to be deficient in HBsAg completion at baseline within our health system. In Cohort 1, 1542 patients and 1568 patients were randomized to the alert and control respectively. In Cohort 2, 2599 patients and 2590 patients were randomized to the alert and control respectively. For both cohorts combined, 389 HBsAg tests were completed in the alert group compared to 177 HBsAg tests in the control group (*p* < 0.0001; OR = 2.3; 95% CI 1.94–2.80), but there was no increased detection of HBsAg positivity from the alert (15 versus 13 respectively, *p* = 0.09; OR = 0.5; 95% CI 0.24–1.09). Our results demonstrate that personalized, automated electronic alerts increase screening for CHB, but more comprehensive measures are needed to detect HBsAg positive patients.

**NIH Trial Registry Number**: NCT04240678.

## Background and significance

Based on the most recent analysis of National Health and Nutrition Examination Survey (NHANES), chronic hepatitis B (CHB) affects 847,000 persons in the United States, which included approximately 400,000 non-Hispanic Asians. These Asians had a tenfold greater prevalence of CHB than the American general population^1^. Foreign-born persons have the highest CHB prevalence in the United States, between 4.5 and 10.3%, and the majority of foreign-born persons with CHB living in the United States originated from Asia^2^. Since CHB is the leading cause of hepatocellular carcinoma and cirrhosis in the world^3^, it is not surprising that Asian and Pacific Islanders (API) living in the US have the highest rates of HCC and HCC-related death^4^.

Because of this, the Centers for Disease Control and Prevention (CDC), United States Preventive Services Task Force, and the American Association for Study of Liver Diseases have recommended screening all persons born in countries with CHB endemicity ≥ 2%. Despite these recommendations, screening rates for CHB remain low, which may be due in part to lack of physician awareness and knowledge about CHB guidelines^5–8^.

With the goal of enhancing clinical decision support related to CHB testing, we have previously published the results of a randomized trial of an electronic health record (EHR)-based alert designed to populate in electronic medical charts of at-risk API with private insurance. We found that the alert caused a more than twofold increase in completion of hepatitis B surface antigen (HBsAg) testing compared to controls after 1 year, but did not increase rates of HBsAg positive tests^9^. At that time, patients with public insurance were excluded due to concerns of costs to patients. Subsequently, Centers for Medicare and Medicaid Services (CMS) began coverage for CHB testing in September 2016^10^ and the cohort for this trial was expanded. Herein, we present the effect of a CHB EHR-alert including patients with private and publicly funded insurance.

## Materials and methods

### Study population

Patients aged 18 years and older with an established primary care provider within the UC Davis health system as of September 17, 2015 with either a self-identified API race or ethnicity or an imputed API race or ethnicity based upon surname, language preference, or country of origin (Supplementary Materials) were enrolled. Patients were excluded if they had prior testing for hepatitis B surface antigen (HBsAg) or an ICD-10 code for chronic hepatitis B (B18.1). Patients meeting any of these criteria for API ethnicity were computer randomized 1:1 to the control group or the alert group which had their electronic medical charts tagged to receive the EHR alert. Surname lists used to identify persons of Asian ethnicities have previously been validated^11–13^.

Two cohorts of patients were analyzed for this study. Because CMS did not initially cover HBsAg testing, Cohort 1 was composed primarily of privately insured patients. Once CMS approved HBsAg testing in September 2016, we were able to randomize Cohort 2 to the alert. The EHR alert was released to this Cohort 1 in January 2016. After CMS approved coverage for HBsAg testing among at-risk individuals, the same EHR alert was released in January 2018 to Cohort 2, which included patients with Medicare or Medicaid insurance. Therefore, Cohort 2 is more representative of the population as a whole compared to Cohort 1. Both cohorts were followed for HBsAg completion until July 2019.

The study was approved by the University of California Davis Institutional Review Board (IRB). Informed consent was waived by the University of California Davis IRB because the study was considered minimal risk and consenting the thousands of patients randomized would not be feasible. All methods were carried out in accordance with relevant guidelines and regulations.

### EHR alert

The deployment of the EHR alert was previously described^9^. It was deployed through the health system’s electronic record (Epic Systems, Verona, WI) under the “Health Maintenance” functionality, which provides reminders for periodic health screenings and preventive care, starting January 28, 2016 to the present day. A completed HBsAg test automatically changed the alert status from “Due” to “Done.” The status could also be changed manually if a patient reported having the test done in another health care system or if a patient refused testing to “previously done” or “patient declined,” respectively. Patients and providers were blinded to the study and no new interventions to increase CHB testing were implemented during the study period. The primary outcome of the study was HBsAg test completion within the health system during the study period. Secondary outcomes included the difference in HBsAg positivity between the alert and control groups.

### Statistical analysis

The statistical methods used were previously described^9^. Proportions between the EHR alert and control groups were compared using Fisher’s exact test. The Wilcoxon rank-sum test was used to compare numerical variables between the EHR alert group and control group. Multivariable logistic regression was used to study the association between receiving the HBsAg test, as a binary outcome variable, and the binary predictor, alert (EHR alert vs. control), in order to adjust for confounders, including insurance, number of office visits, age, sex, and language. A *p* value < 0.05 was considered statistically significant. Statistical analyses were performed with SAS v 9.4 (SAS Institute Inc., Cary, NC, USA).

## Results

**Figure 1:**
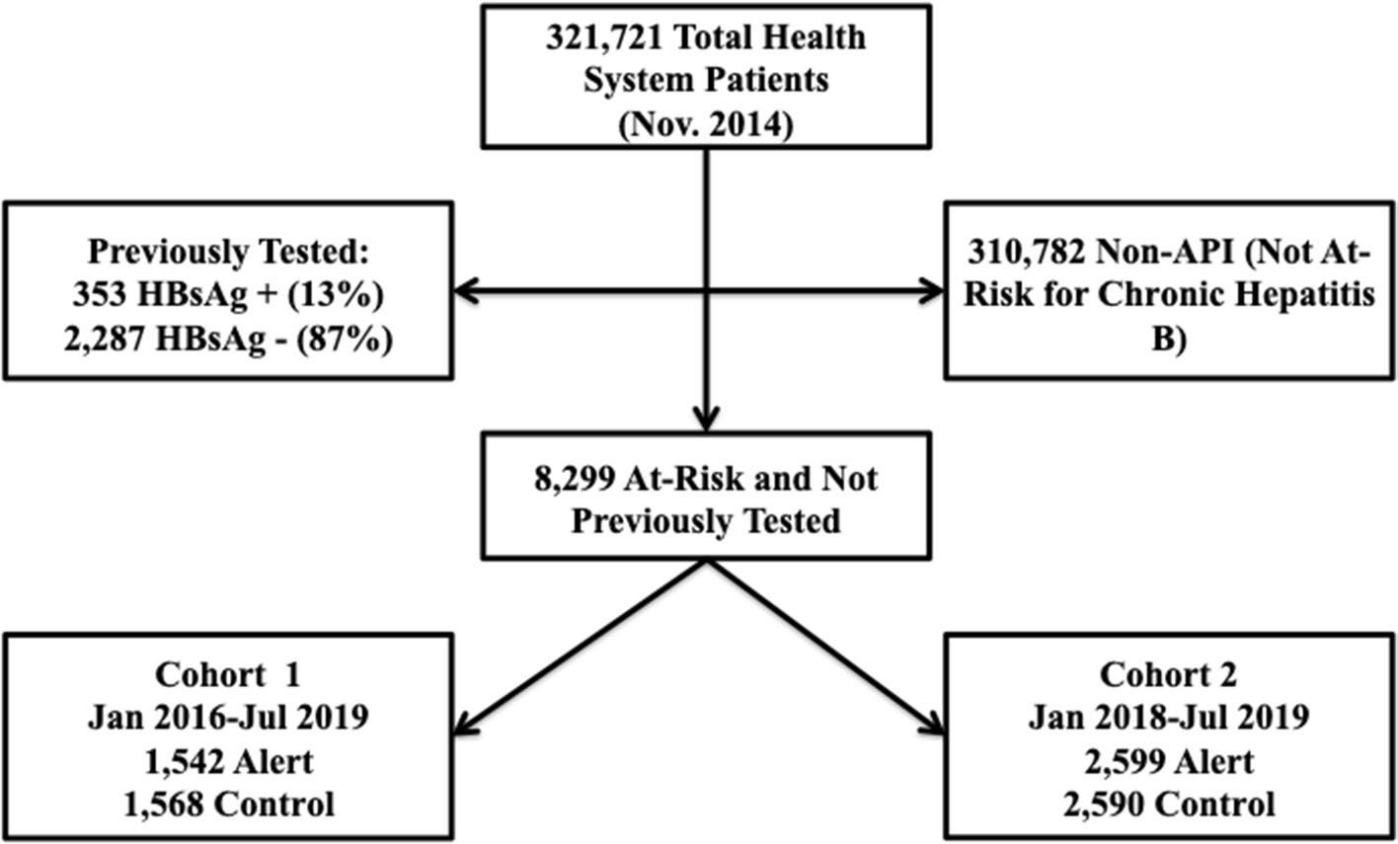
CONSORT diagram for patients in the HBV screening alert study. A total of 8299 at-risk patients were determined to have never completed HBsAg testing and were randomized for the study.

In January 2016, the total number of patients within the health system at study initiation was 321,721. Of these, 2640 had documentation that they had been previously tested. 353 (13%) were HBsAg positive, 2287 (87%) were HBsAg negative, and 8299 were API who had not yet completed HBsAg testing (Fig. 1). Cohort 1, implemented prior to CMS coverage of HBsAg testing, included 3110 patients of which 78% had private insurance. There were no significant baseline differences between the alert and control groups (Table 1). Cohort 2, which included Medicare and Medicaid, consisted of 5189 individuals, of which 55% also had private insurance (Table 2). There were no significant baseline differences between the alert and control groups in either Cohort 1 or 2.

**Table 1:** Baseline characteristics cohort 1 (January 2016–July 2019).

Characteristic	Alert (N = 1542)	Control (N = 1568)	*p* Value
Male (%)	739 (47.92)	712 (45.41)	0.16
English primary language (%)	1214 (78.73)	1244 (79.34)	0.69
Age (mean years ± SD)	42.65 ± 14.66	43.04 ± 14.78	0.49
Insurance (%)			0.55
Private	1201 (77.89)	1211 (77.23)	
Medicare	34 (2.20)	48 (3.06)	
Medicaid	9 (0.58)	12 (0.77)	
Self-pay	288 (18.68)	284 (18.11)	
Other	10 (0.65)	13 (0.83)	
# Office visits (mean ± SD)	7.00 ± 7.16 (N = 668)	7.70 ± 9.51 (N = 675)	0.81

**Table 2:** Baseline characteristics cohort 2 (January 2018–July 2019).

Characteristic	Alert (N = 2599)	Control (N = 2590)	*p* Value
Male (%)	1236 (47.56)	1185 (45.75)	0.20
English primary language (%)	1805 (69.45)	1810 (69.88)	0.74
Age (mean years ± SD)	51.64 ± 20.88	51.32 ± 20.66	0.67
Insurance (%)			0.95
Private	1420 (54.64)	1418 (54.75)	
Medicare	502 (19.32)	493 (19.03)	
Medicaid	357 (13.74)	366 (14.13)	
Self-pay	310 (11.93)	300 (11.58)	
Other	10 (0.38)	13 (0.50)	
# Office visits (mean ± SD)	4.77 ± 4.73 (N = 756)	5.4 ± 6.3 (N = 743)	0.09

Regarding the effect of the EHR alert on HBsAg testing in Cohort 1, over the course of 3 years, 269 of 1542 (17.4%) completed HBsAg testing in the alert group compared to 133 of 1568 (8.5%) (*p* < 0.0001; OR = 2.3; 95% CI 1.83–2.84,) in the control group. This rate of HBsAg completion increased from 8% (after 1 year) to 17.4% (after 3 years). Of those who completed HBsAg testing in Cohort 1, 4.1% were positive in the alert group versus 7.5% in the control group (*p* = 0.16) (Table 3). The percentage of HBsAg positive individuals identified in the alert and control groups were 0.7% and 0.6%, respectively.

**Table 3:** Effect of HBV EHR alert on HBsAg test completion (cohorts 1 and 2).

Cohort 1 (January 2016 to July 2019)				
HBsAg alert status	Alert (N = 1542)	Control (N = 1568)	OR (95% CI)	*p* Value
HbsAg completed (%)	269 (17.4)	133 (8.5)	2.3 (1.83, 2.84)	< 0.0001
HbsAg positive (%)	11 (4.1)	10 (7.5)	0.52 (0.22, 1.27)	0.16

Cohort 2 (January 2018 to July 2019)				
HBsAg alert status	Alert (N = 2599)	Control (N = 2590)	OR (95% CI)	*p* Value
HBsAg completed (%)	120 (4.6)	44 (1.70)	2.80 (1.97, 3.98)	< 0.0001
HBsAg positive (%)	4 (3.3)	3 (6.8)	0.47 (0.10, 2.20)	0.39

In Cohort 2, 120 of 2599 (4.6%) completed HBsAg testing in the alert group compared to 44 of 2590 (1.7%) in the control group (*p* < 0.0001; OR = 2.8; 95% CI 1.97–3.98). Of those who completed HBsAg testing in Cohort 2, 3.3% were positive in the alert group compared to 6.8% in the control group (*p* = 0.39) (Table 3). In Cohort 2, the percentage of HBsAg positive individuals identified in the alert and control groups was 0.2% and 0.1%, respectively. Overall, the rate of HBsAg positivity for both cohorts combined was 28 HBsAg positives of 566 HBsAg tests completed (4.9%).

**Figure 2:**
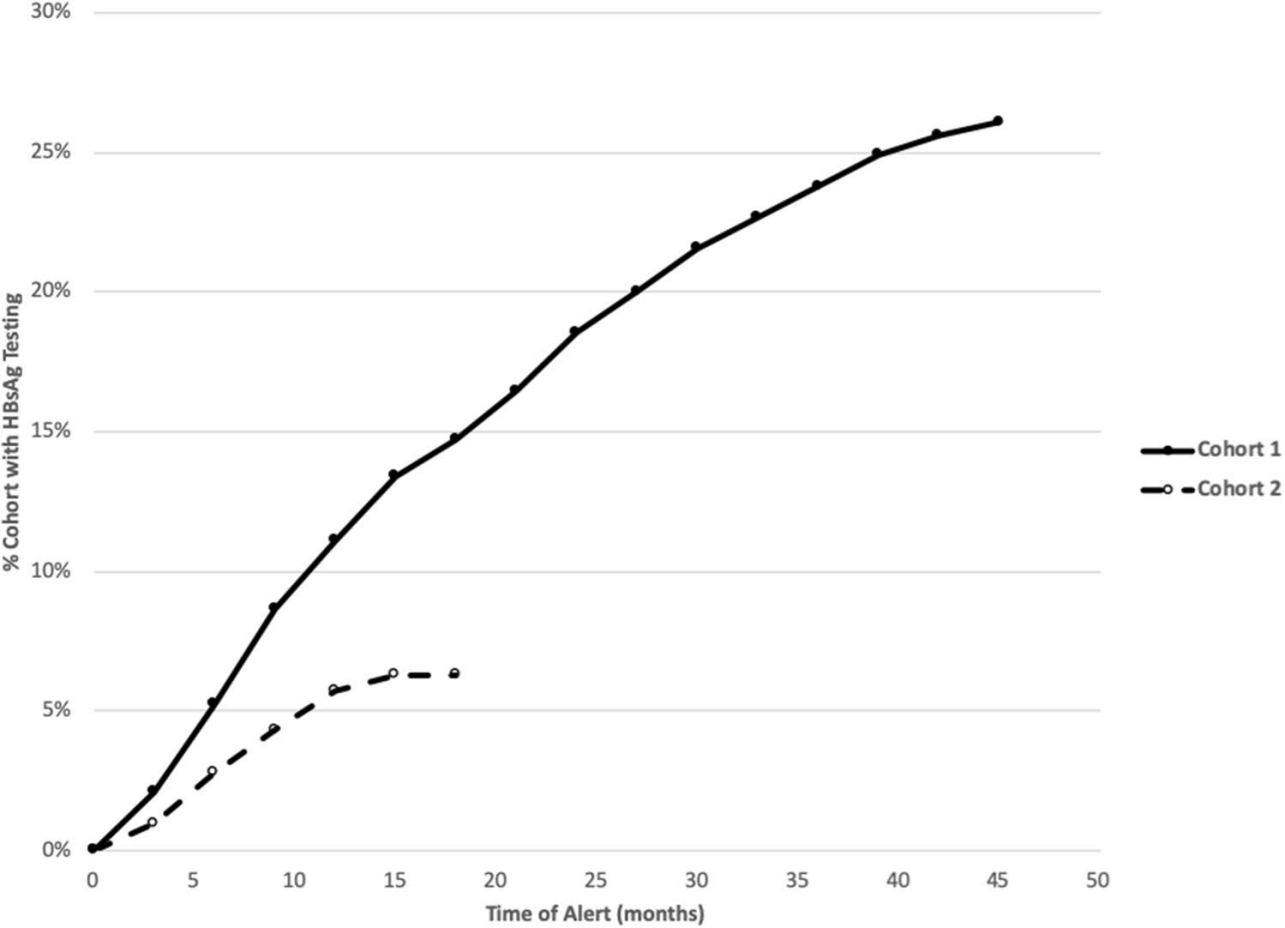
HBsAg completion among API in the alert and control groups. The figure shows percentage of HBsAg completion in each cohort related to time after randomization to alert or control.

Because testing is more likely to occur with an office visit, we performed an analysis limited to those patients who attended at least 1 office visit during the study period. Among this group, HBsAg test completion similarly favored the alert group with 358 of 1424 patients (25.1%) in the alert group completed a HBsAg test compared to 154 of 1418 patients (10.9%) in the control group (*p* < 0.0001, OR 2.8; 95% CI 2.4–3.4) with no difference in HBsAg positive tests (12 alert vs 10 control, *p* = 0.09). Regarding the number of HBsAg tests completed over the study period, there appeared to be a decrease in HBsAg completion rate over time, which is more pronounced starting Quarter 1 of 2019 and onward (Fig. 2).

Multivariable logistic regression analysis showed there was a statistically significant association between HBsAg test completion and the alert (*p* < 0.0001; OR = 3.23; 95% CI 2.24–4.67), number of office visits (*p* < 0.0001; OR = 1.13; 95% CI = 1.10–1.16) and age (*p* = 0.03; OR = 1.01; 95% CI = 1.00–1.02). HBsAg test completion was not affected by sex or language preference.

In Cohort 2, 3.6% (103 of 2838) of privately insured and 5.1% (51 of 995) of Medicare patients completed HBV testing compared to 0.6% (4 of 723) of Medicaid patients and 0.8% (5 of 610) of self-paying patients. Multivariable logistic regression analysis showed that patients with private and Medicare insurance were more likely to complete HBV testing compared to Medicaid (*p* = 0.001 and 0.01; OR = 5.176; 95% CI = 1.887, 14.197 and OR = 4.536; 95% CI 1.561, 13.181 respectively). Similarly, patients with private and Medicare insurance were more likely to complete HBV testing compared to those who self-pay (*p* = 0.01 and 0.03; OR = 3.40; 95% CI 1.369, 8.447 and OR = 2.980; 95% CI 1.110, 7.996 respectively) (Table 4).

**Table 4:** Multivariable logistic regression analysis of characteristics associated with HBsAg test completion.

Characteristic	aOR (95% CI)	*p*
Insurance		
Private vs. medical	5.176 (1.887, 14.197)	0.001
Medicare vs. medical	4.536 (1.561, 13.181)	0.01
Private vs. self-pay	3.400 (1.369, 8.447)	0.01
Medicare vs. self-pay	2.980 (1.110, 7.996)	0.03
Alert (EMR alert vs. control)	3.233 (2.240, 4.666)	< 0.0001
Number of office visits	1.131 (1.102, 1.160)	< 0.0001
Age	1.012 (1.001, 1.022)	0.03
Sex (male vs. female)	1.294 (0.935, 1.791)	0.12
Language (English vs. non-English)	1.217 (0.837, 1.769)	0.30

^a^aOR: model controls for alert status, office visit, age, sex, and language.

## Discussion

In this 43-month double-blind, randomized trial, we found that an automated EHR-based alert increases CHB testing in API individuals by more than twofold. Of those who completed HBsAg testing, 4.9% were positive. Thus as a proof of concept, we have shown that EHR-based alerts provide incremental benefit towards increasing CHB screening. We have previously shown that electronic messages to health care providers placed within 24 h prior to a clinic visit were effective at increasing CHB testing for at-risk individuals. However, this approach was resource intensive, could not be easily automated, and interrupted workflows^14^. In this regard, the automated alert described herein is an improvement on our previous method.

Automated electronic medical alerts have been validated as clinical decision support tools in a variety of disease states^15–18^. However, unlike chronic hepatitis C or other common preventive health care measures where screening is based on age and is accurately captured in the EHR, mass screening for CHB requires more personalization as it is based upon country of nativity, which is typically not recorded in the EHR. Thus, we used a novel approach to identify patients who were at risk for CHB using imputed API race or ethnicity based upon surname, language preference, or country of origin. Additional strengths of this study include its randomized design and length of blinding. Many similar studies measure the impact of EHR interventions before and after implementation, which can be confounded by other changes in practice and knowledge over time. The nearly 4 years of implementation of the alert in Cohort 1 also demonstrates that there is a potential for saturation of an alert and that uptake may not continue in a linear fashion. Interestingly, saturation appeared to have been reached much more rapidly in Cohort 2, which may be due to alert “burn out” among healthcare providers.

Results of multivariable logistic regression analysis showed that patients with Medicaid insurance or those who self-pay for medical care had lower odds of completing HBV testing compared to privately insured patients and those with Medicare. This healthcare access disparity may explain the lower than expected detection of HBsAg positives in the alert group. In theory, patients with Medicare and Medicaid should be at higher risk for CHB. Medicare patients are older and less likely to have been vaccinated against hepatitis B (HBV) since universal infant vaccination in the United States began in 1982^19^. Medicaid patients likewise may have decreased access to medical care, perinatal HBV screening, and HBV vaccination. Despite inclusion of these higher risk groups, we did not find an increased number of HBsAg positives in the alert group.

Of the 4141 total patients in the alert group (Cohort 1 and 2 combined), only 389 (9.4%) completed HBsAg testing so while the alert did increase the chance of HBsAg completion, the overall effect was small.

There were limitations to the CHB alert and the current study. The first limitation is that the alert is passive. That is, it lays dormant in the medical chart of the at-risk person until he or she visits their physician. Only after the physician opens the medical chart and discovers the alert, can the effect of the alert be manifest. However, even among patients that attended an office visit during the study period, a similar twofold increase in HBsAg testing without an increase in HBsAg positive detection was found. Multivariate analysis did show that both the alert and attendance of office visits were independently associated with completion of HBsAg testing. More active measures to engage and educate at-risk patients are needed to bring them to their physicians for screening. The second limitation to the CHB alert is that some API may not have been identified in the population. Our algorithm utilizes surnames that are associated with API ethnic groups but excludes surnames that are ambiguous for API ethnicity, for example “Lee”. Adopted APIs, APIs that have taken on a non-API married surname, or non-APIs that have taken on API surnames may also lead to misclassification. Third, we did not screen other risk groups such as African born, persons who inject drugs, and men who have sex with men. Fourth, since CMS did not initially cover HBsAg testing, we needed to use 2 Cohorts. This may have decreased the effectiveness of the alert in Cohort 2, due to contamination. That is, PCP awareness of hepatitis B testing may have increased during Cohort 1 and more tests could have been ordered regardless of alert status during Cohort 2.

In conclusion, EHR alerts increase completion of HBsAg testing, but do not increase detection of HBsAg positive cases even over years of follow up, but this may be explained by differences in insurance status. Screening for CHB requires a personalized approach since the decision to screen is based upon country of birth. While electronic alerts may have a role in increasing CHB screening, they will likely be ineffective alone. A multifaceted approach involving patient outreach and engagement to draw at-risk patients to CHB screening opportunities is needed.

## Supplementary information

Supplementary Information 1.

Supplementary Information 2.
