# Supplementary material for: Electronic health record alerts enhance mass screening for chronic hepatitis B
Source: Sci Rep. 2020 Nov 5;10:19153. doi: 10.1038/s41598-020-75842-8 (PMC7644717; doi:10.1038/s41598-020-75842-8)
Supplement: Supplementary file 2 — Supplementary Information 2. [file 41598_2020_75842_MOESM2_ESM.docx]

**Supplemental Material Title: Surname List Used to Identify Asians for Hepatitis B Alert**

**Manuscript Title: Electronic Health Record Alerts Enhance Mass Screening for Chronic Hepatitis B**

Authors: Eric Chak^1^, Chin-Shang Li^2^, Moon S. Chen, Jr.^3^, Scott MacDonald^4^, Christopher Bowlus^1^

Affiliations:

^1^UC Davis School of Medicine, Division of Gastroenterology and Hepatology,

Sacramento, California

^2^School of Nursing, The State University of New York, University at Buffalo, Buffalo, NY

^3^ UC Davis School of Medicine, Division of Hematology and Oncology, Sacramento, California

^4^UC Davis Medical Center, Division of Clinical Informatics, Sacramento, CA
